# A Biological Approach to Antimicrobial Resistance in New and Old Bacterial Pathogens

**DOI:** 10.3390/pathogens15090977

**Published:** 2026-09-15

**Authors:** Robert J. Atterbury, Adriano M. Gigante, Paul A. Barrow

**Affiliations:** 1 School of Veterinary Medicine and Science, University of Nottingham, Sutton Bonington Campus, Sutton Bonington, Leicestershire LE12 5RD, UK; 2 School of Veterinary Medicine, University of Surrey, Daphne Jackson Road, Guildford GU2 7AL, UK

**Keywords:** antibiotics, antibiotic resistance, antimicrobial resistance, bacteriophage, plasmid

## Abstract

Resistance to antimicrobials in bacterial pathogens has become a major global public health issue that international institutions are now realizing must be tackled with a degree of urgency. A major issue is the self-transmissibility of the plasmids that carry most of the resistance genes. Scientists are now exploring ways in which the antimicrobial resistance plasmids might be tackled directly to reduce resistance. Pharmacological approaches to destabilizing plasmids have not yet been tested in vivo. Phagemids may be used to introduce genes which harm the bacteria themselves or disrupt key plasmid genes. The transfer of transmissible plasmids is mediated by thin conjugative pili which are also attachment sites for a range of lytic bacteriophages. Such phages may be used to kill bacterial cultures where fimbriae are highly de-repressed. They also select for the small number of bacterial cells which have spontaneously lost their plasmid, thereby replacing an antibiotic-resistant strain with one which is once again fully susceptible to antibiotics. This has been demonstrated both in vitro and in animal models of infection. A pharmaceutical approach is proposed to de-repress the plasmids possessed by many strains and thereby increase phage susceptibility.

## 1. AMR as an Existential and Global Problem

Antimicrobial resistance (AMR) is now regarded as an existential threat to human and animal health [1]. Widespread AMR results largely from both regulated and unregulated use of antimicrobials for therapy and prophylaxis in human and veterinary medicine, for metaphylaxis in food animals, and from unregulated use for growth stimulation of livestock over many decades since the 1940s, and which continues to occur in many parts of the world.

Human deaths attributable to AMR are already estimated to be greater than 1 million p.a. [2,3]. AMR-associated mortality could reach 10 million p.a. by 2050, affecting global GDP by 2–3.5%. All infections involving AMR pathogens incur higher hospital and treatment costs [4,5].

Levels of AMR are high in major pathogens and commensals including *E. coli* and many opportunistic pathogens including the ESKAPE pathogens [6], namely *Enterococcus faecium*, *Staphylococcus aureus*, *Klebsiella pneumoniae*, *Acinetobacter baumannii*, *Pseudomonas aeruginosa*, and *Enterobacter*. Additional critical priority bacterial taxa responsible for opportunistic infection include other members of the Enterobacteriales such as *Serratia*, *Proteus*, *Providencia* and *Morganella* [7]. The significance of these opportunistic pathogens lies in their association with serious clinical disease in hospital patients or those who may be immunocompromised.

Much of the AMR present in these bacteria, including Gram-positive organisms such as *E. faecium* [8], is also transmissible, involving self-transmissible plasmids possessing transposons and integrons [9], although many clinically important resistance determinants are also chromosomal. The AMR plasmids of some Multiple Drug Resistant (MDR) clones such as *E. coli* ST131 increase bacterial fitness, which has facilitated global spread [10]. Pathogens such as *A. baumannii* [11], *K. variicola* [12] and *P. aeruginosa* are essentially environmental bacteria which may also be found on human skin and have been isolated from clinical equipment, and thus represent huge potential clinical pathogens. Other more recently reported AMR pathogens may infect man as food-borne pathogens, including *Aerobacter*, *Cronobacter* and *Alciabacter* [13]. The clinical environment is one where antibiotics and other anti-bacterial compounds, heavy metals and disinfectants are present, which can also select for AMR bacteria. Food-borne pathogens or commensals may have had contact with antibiotics during livestock treatment with an estimated 70–80% of antibiotic use being in agriculture [14].

National governments and international institutions [15,16,17] have called for a comprehensive strategy, with tighter national regulation, improvements in diagnosis, the identification of new drugs, and the development of new and innovative approaches to tackling the problem. Existing proposed measures together with improvements in animal husbandry, including biosecurity, vaccine use and probiotic development, will undoubtedly lead to reductions in AMR. However, they will not have an immediate positive effect on existing infections caused by bacteria which are already resistant. The development of new small-molecule antimicrobials targeting essential microbial processes [18] has led to very few real outputs. Bacteriophages (viruses that infect bacteria) have been identified specifically as one of several novel approaches to combating AMR [15,17,19,20].

## 2. Bacterial Plasmids and AMR

Much of the clinical, epidemiological and evolutionary significance of AMR for both Gram-negative and -positive bacteria lies in its transmissibility.

Most AMR genes in *E. coli* and related bacteria are located on self-transmissible plasmid-containing genes encoding proteins facilitating their own replication and self-transmissibility. Genes contributing to bacterial fitness and survival include resistance to antimicrobials, with some plasmids encoding resistance to up to 10–20 antibiotics simultaneously (including antibiotics of last resort, such as polymyxins and carbapenems) [21]. Plasmids can also encode for resistance to heavy metals, but can also encode a variety of virulence factors, bacteriocin production and degradative metabolic functions. Much AMR in Gram-positive bacteria is also mediated by transmissible elements such as transposons and integrons which are integrated into plasmids and thus enhance their transmissibility. Plasmids ensure their own maintenance and avoid segregation during cell division by a variety of mechanisms, including killing daughter bacterial cells which do not inherit a copy of the plasmid. There is a variety of suicide and Toxin/Antitoxin (TA) systems [22].

Plasmids are classified into Replicon/Incompatibility (Inc) groups so that plasmids belonging to the same group cannot stably coexist in the same bacterial cell. Because the copy number of wild-type plasmids is generally tightly regulated (for some large plasmids the copy number is only 1–2 per cell), the existence of two different plasmids belonging to the same Inc group usually results in segregation at cell division. A large number of replicon/Incompatibility (Inc) types exist. The first plasmid discovered, the F (fertility) factor in 1952, belongs to the IncF group. This group is the most frequently encountered Inc group in human AMR and is also found frequently amongst AMR bacterial strains in animals [21,23,24].

Amongst IncF plasmids, self-transmissibility is mediated mainly by a single large transfer operon (the *tra* operon), 33 kb in size and encoding > 40 genes involving a single transcribed RNA species [25]. The *tra* operon mediates mating pair formation, DNA replication and transfer to compatible recipient bacteria together with other features such as surface exclusion. A protein—pilin—coalesces into a long adhesive sex pilus which attaches to the recipient cell and which also acts as a receptor for selected bacteriophages. IncF plasmid pili are receptors for icosahedral lytic Emesviruses (formerly Leviviruses) and filamentous Inoviruses, the latter of which are not lytic, and there is a degree of specificity with phages specific for the sex pili produced by different Inc group plasmids [26].

## 3. What Can Be Done About AMR Plasmids—Introduction

The approaches advocated for in the following sections aim to reduce the prevalence of AMR bacteria by targeting and eliminating/curing the plasmid and AMR genes rather than the bacteria themselves, thereby restoring susceptibility to antibiotic chemotherapy. This approach also broadens the range of bacterial species which can be targeted since, if phages are used, the attachment to most surface antigens as receptors is generally very specific to a bacterial species or even serotype, whereas a plasmid producing the same sex pili can be possessed by a range of different bacteria.

In the absence of antimicrobials, plasmid-free bacteria generally show greater fitness than bacterial cells possessing a plasmid and, therefore, will usually outcompete the plasmid-containing AMR strains. However, the ecological dynamic between plasmids and their hosts is complex and dependent on various environmental, ecological and genetic factors [27]. Plasmid-free bacterial mutants also represent potential recipients into which AMR plasmids could re-enter, but rates of conjugation in the gut containing normal microbiota are very low, at least in the absence of antibiotics [28,29]. For acute infections, administration of an agent inducing plasmid-curing (loss) simultaneously with chemotherapy involving an antibiotic to which the bacterial strain was previously resistant is an extremely attractive scenario. The methods currently being explored have all been demonstrated to be effective in vitro with a smaller number tested in animal models. None, as far as we know, has yet been assessed in a full clinical setting.

## 4. What Can Be Done About AMR Plasmids

### Pharmaceutical Exploration of Plasmid Stability

This has been reviewed several times [30,31,32,33,34]. Plasmid maintenance and survival require several functions which may be interfered with. Aminoglycoside antibiotics disrupt plasmid replication and induce elimination [22]. Further work has tried to identify the thermodynamic characteristics of small-molecule binding to search for non-antibiotic candidates [34], because it is clearly inappropriate to use an antibiotic to eliminate an AMR plasmid. Using a plasmid-based induction of incompatibility, interfering with segregation, can result in reduced intestinal colonization of mice by AMR *E. coli* strains [35]. However, this only occurred when the mice were treated orally with an antibiotic which would increase in vivo conjugation rates by reducing the normal microbiota.

Toxin–antitoxin (TA) systems generally function by way of the antitoxin being less stable than the toxin or by a small intracellular molecule disrupting the toxin–antitoxin binding [22]. These systems have been explored, but primarily as a means to reduce bacterial viability [36,37]. The potential harmful effects of toxin release into the eukaryotic hosts have been raised as a concern [36]. Similar approaches, with a toxin-producing gene, such as colicin E, introduced into a recipient cell from a donor expressing a repressor for the toxin, have been explored to restrict the spread of recombinant DNA [38,39].

These approaches [40] will certainly kill AMR bacteria but not replace the AMR cells by AMS derivatives, which could be seen as the ideal objective. An advantage of using systems such as those above is that plasmid-curing can be obtained with plasmids showing any level of repression (vide infra). However, it is necessary that the conjugation of the donor plasmid is extensive enough in vivo because of the low in vivo rates of conjugation [28,29].

In vitro screening for inhibitors of conjugation has identified linoleic acid, bisphosphonates and dehydrocrepenynic acid (DHCA), in addition to several others which are toxic to eukaryotes [32]. The mechanism of action of bisphosphonates is unclear [41], but they have an advantage in that they are already in use for treatment of bone disease. Intrabodies are antibody fragments (Fv) which are expressed within the bacterial cell and which have been used to target the relaxase [42].

These approaches have not been evaluated in vivo.

## 5. What Can Be Done About AMR Plasmids?

### 5.1. Bacteriophages—Introduction to Lytic Bacteriophages as an Antibacterial Remedy

Lytic bacteriophages (phages) were proposed for use in human and veterinary medicine soon after their discovery in the early 20th century. A combination of an inadequate understanding of their nature and of bacterial pathogenesis in that period, as well as poor experimental design and the advent of antibiotics, led to their neglect in the West by the 1950s, although they continued to be used in Eastern European countries. Interest was resurrected in the 1980s after some high-quality experimental work in animals [43,44,45], showing that they could be highly effective and even more effective than antibiotics [43,45,46]. This has led to a resurgence of interest summarized by Chang et al. [47]. Bacteriophages have been assessed extensively in vivo, including animal experiments with ESKAPE pathogens. Similarly, a number of clinical trials have been carried out or are under way to assess efficacy in human medicine [47]. The increasing use of phages has led to a recently developed roadmap for their further exploitation [20].

There are advantages and also disadvantages in using bacteriophages for disease therapy. Advantages include the fact that phages multiply naturally in the host and repeated doses may not be necessary. They have minimal effect on the microbiome, unlike most antibiotics. They are reported to be effective against biofilms, although in some cases, as particulate entities, they are likely to be less effective than a chemical which can diffuse more easily. They are also relatively cheap to produce.

One major disadvantage is that they are exquisitely specific, sometimes to strains within a species. For a Magistral preparation [48], this is acceptable and indeed desirable, but for an acute infection where there has not been time for the bacterial strain to be tested for phage sensitivity this is a clear disadvantage. They are also immunogenic, although for acute infections and short-term treatment this is likely to be less of a problem. Certainly, in some trials [45,49] circulating neutralizing antibodies were not detected in experimentally infected and phage-treated chickens. Phages can these days be checked for the absence of harmful genes so that the transduction and dissemination of virulence genes picked up in the host strain for multiplication should not be a problem. Similarly, temperate phages can be manipulated to remove integrase and repressor genes to convert into fully lytic phages, removing risks of chromosomal integration.

Many phages use surface structural components such as lipopolysaccharide as receptors, which can, however, limit the range of targeted bacteria in a clinical setting. An additional key negative feature of phage therapy is the development of phage resistance during treatment. This can be addressed by using combinations of phages that cover the range of bacterial strains and species expected in an infection [50].

The development of resistance to the phage may also be reduced by using a combination of phages, one which targets the initial pathogen and a second which targets the phage-resistant mutants arising during treatment by the first phage [44,46,51]. An alternative approach is to use phages that target surface virulence determinants such that most phage-resistant mutants would be less virulent [43,45]. This is an approach that we have adapted for AMR bacteria using phages that attach to the sex pili produced by self-transmissible plasmids as phage receptors (vide infra).

The use of phages combined with antibiotics has been explored to reduce this problem and, although not specifically as a means to reduce AMR, this is an outcome. There does seem to be a degree of synergy between phage and simultaneous chemotherapy in broth, in vitro and in vivo, although this is dependent on whether the antibiotic concentrations are above or below the MIC [52,53,54,55,56,57,58,59,60]. Biological material, such as urine and serum, can reduce any synergistic effects [56]. The published work has involved a variety of phages and bacterial species (including the ST131 multi-antibiotic-resistant global clone of *E. coli*), and also *Candida albicans* [59,60]. The greatest effects appear to be with combinations of phage and β-lactam antibiotics with the antibiotics adversely affecting cell wall structure and also leading to filamentation, which increases phage activity [61]. The nature of the antibiotic and which cellular components are affected also determines whether the combinatorial effects are synergistic, additive or produce negative effects or no effect at all [58]. Combinations of phages and antibiotics may also reduce the development of phage resistance during treatment by suppressing the growth of secondary mutants [61].

### 5.2. Engineered Phages and CRISPR-Cas Gene Inactivation Systems

Apart from the use of native bacteriophages, phage genes may be manipulated to facilitate additional beneficial features in terms of intra-bacterial cellular activity, host-specificity, etc. [62]. Some small RNA phages might be synthesized de novo, which could make screening for altered receptor specificity easier.

The use of phagemids, which combine features of plasmids and filamentous phages, has been particularly useful combined with the CRISPR-Cas precise mutation system. Since its initial discovery in the late 1980s, the CRISPR-Cas system has become an essential tool for rapid bacterial mutation and gene editing in both bacteria and eukaryotic cells [63]. With the capacity to produce sequence-specific lethal double-stranded DNA breaks, they have also been explored as a means to reduce bacterial viability and manipulate bacterial virulence. This included the elimination of *srtA* expression in *Staphylococcus aureus* [64] and *stx* in an EHEC strain of *Escherichia coli* [65]. Phagemids can also be adapted to deliver toxic proteins to bacterial cells. This can be useful when the bacterial death is not sought. Toxin of the toxin–antitoxin systems can be delivered, which can be lethal to the bacterial cells [66]. The technique has been studied extensively in vitro to eliminate AMR gene expression, including *mecA* in *S. aureus* [67], and in a carbapenem-resistant strain of *Klebsiella pneumoniae* [68] in addition to other ESKAPE pathogens [69]. Temperate phages have also been engineered to incorporate CRISPR/Cas into their genome [70] and increase their host range by tail fiber manipulation, and have been used to reduce skin colonization in mice by *S. aureus* [71].

In vivo delivery will always be an issue with the current inability of phagemids to spread through a bacterial population without a helper phage. The use of f1/m15 currently also restricts their use to bacteria harboring de-repressed AMR Inc F plasmids or Hfr strains, and although IncF plasmids are dominant in human medicine, most plasmids show repression of transmissibility and sex pilus production (vide infra).

Conjugation methods have been used extensively for the delivery of plasmids encoding CRISPR-Cas genes to recipient bacteria. Rodrigues et al. [72] used a pheromone-responsive plasmid to deliver Cas9 into *Enterococcus faecalis*, reducing erythromycin and tetracycline resistance with the added provision of bacteriocin production by the plasmid to kill recipient bacterial cells that had not received the plasmid. Low conjugation rates were observed in the intestines of dysbiotic mice treated with antibiotics. Low rates of antibiotic-resistant gene loss were observed in vitro, and although not checked, in vivo bacterial numbers were reduced. Zhou et al. [73] showed plasmid-curing from a resistant strain of *K. pneumoniae* in *Galleria mellonella* and increased survival of the host. Sheng et al. [74] also used conjugation to deliver Cas9 into a β-lactamase resistant *E. coli* O55:H9 in neonatal mice, showing good reduction in bacterial numbers. Because in vivo rates of conjugation are generally low [28,29] in the absence of initial antibiotic treatment, delivery with nanoparticles has also now been considered [75]. Low conjugation rates in the intestine may also affect CRISPR-Cas approaches to modifying microbiome components using conjugation to introduce modifying plasmids into target bacteria [76].

### 5.3. Other Biological Systems

In addition to lytic bacteriophages, a small number of other biological systems, including parasitic bacteria and endolysins, are available, but which target the bacteria whether or not they are antibiotic-resistant. *Bdellovibrio bacteriovorus* is an endoparasitic bacterium that is able to predate a wide range of Gram-negative bacteria. There have been a number of demonstrations of in vivo efficacy, including reducing bacterial numbers in a lung infection in mice by *K. pneumoniae* [77], reducing respiratory infection in mice by *Yersinia pestis* [78], and reducing intestinal colonization in chickens by *Salmonella* Enteritidis [79].

Endolysins are proteins produced by bacteriophages for release from the bacterial cell, following vegetative reproduction. They are extremely effective against Gram-positive bacteria since they act directly on the peptidoglycan. The presence of the outer membrane in Gram-negative bacteria makes this group more intractable, but the use of permeabilizers such as EDTA can improve access to the cell wall. Endolysins have been found that can target both Gram-positive and -negative bacteria [80]. A new generation of endolysins is being developed to address the clinical conditions under which they may be applied and to reduce inflammatory responses [81]. A number of trials are underway assessing endolysins against superficial Gram-positive infections [82], but there is some consensus that, with ambiguous clinical results, their use might be most effective in association with other chemotherapeutics [83].

## 6. Bacteriophages Specific for Chromosomal and Plasmid-Encoded AMR

There are two major phage-based strategies being explored to address the specific issue of AMR. The advantage for both strategies is that the phages target major components of the AMR mechanism, so the specificity is for the AMR, not the bacteria, effectively broadening host specificity.

### 6.1. Targeting Antibiotic Resistance via Efflux Pumps

This approach targets drug-resistance mechanisms, albeit those which are not necessarily specifically plasmid-associated. Chan et al. [84] isolated a Myoviridae phage OMKO1, for which the receptor was the OprM porin from the MexAB and MexXY drug efflux pump system in a multi-drug-resistant strain of *Pseudomonas aeruginosa*. Phage-resistant mutants developed after phage activity in vitro. This action resulted in increased susceptibility to a range of antibiotics, resistance to which was mediated by efflux activity.

### 6.2. The Interaction Between Male-Specific Phages and AMR Plasmids

The relationship between bacterial fertility and susceptibility to particular phages has been known for decades. These phages are specific to the plasmids rather than to the bacteria. A wide variety of icosahedral and filamentous phages specific for the pili, produced by plasmids of different Incompatibility groups, have been identified and were characterized in the 1980s [26], although many of these are not now available to science.

The specificity of individual phages for Incompatibility groups in previous studies is not absolute, which may be relevant to their application in plasmid-curing/elimination. The use of icosahedral phages (Tectiviridae, including PRD1, and Emesviridae, formerly Leviviridae, including MS2) for this purpose has been reviewed recently [26,85,86,87,88,89].

**Figure 1:**
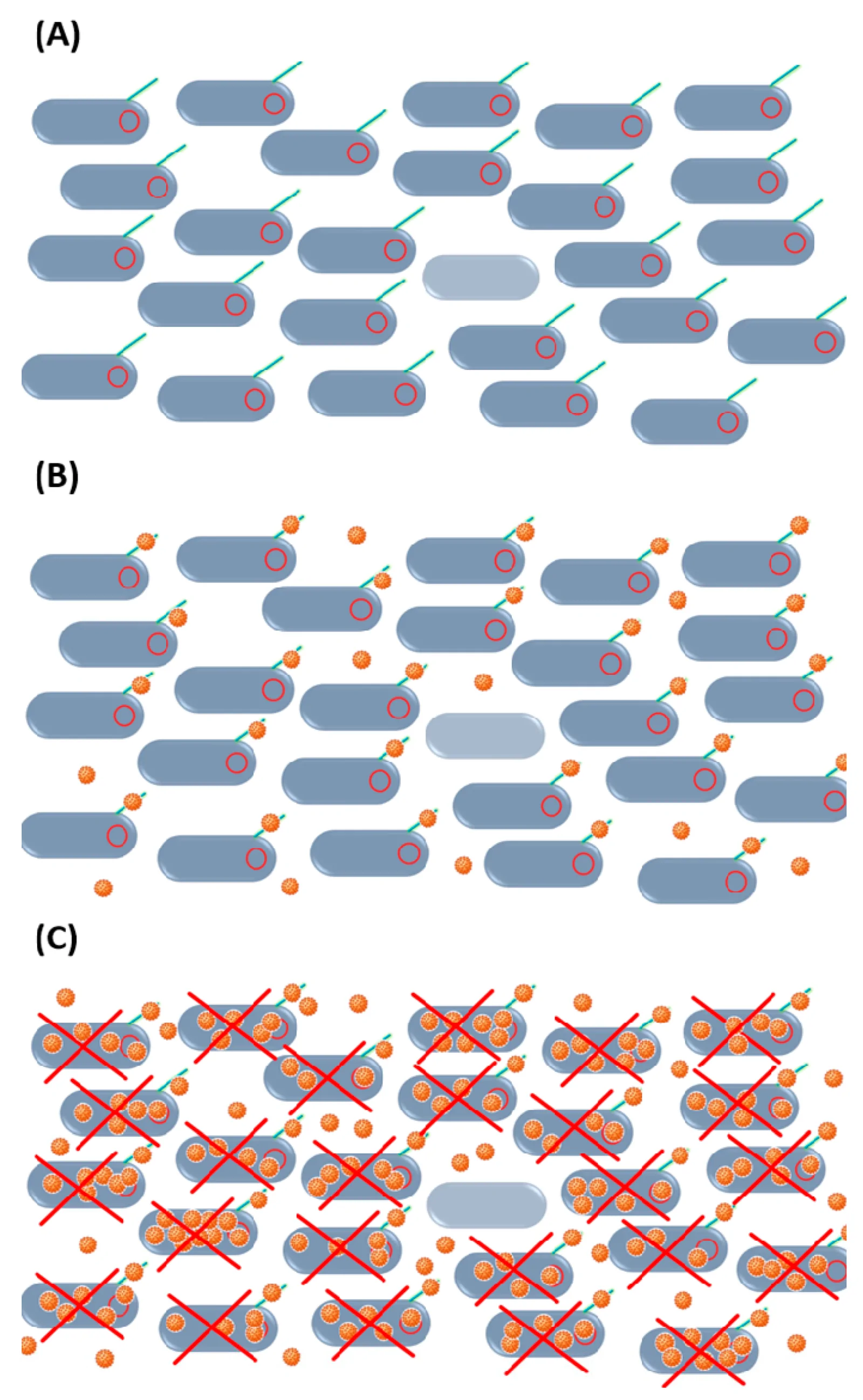
Putative mechanism of phage-mediated plasmid elimination. (**A**) A very small proportion of bacterial cells harboring self-transmissible plasmids will have lost their plasmid despite the presence of toxin–antitoxin (suicide) systems, and the cells do not therefore produce sex pili, which are the receptors for sex pilus/male-specific bacteriophages. One bacterial cell is shown in this state. (**B**) When cultured in the presence of high-titer bacteriophages, phages attach to nearly all the bacterial cells possessing plasmids. The few bacterial cells without plasmids are resistant to attack. (**C**) For highly self-transmissible plasmids the phages kill and lyse the bacterial cells, which possess the plasmids, releasing further phages. The plasmid-free bacterial cells are now able to grow and multiply freely.

**Figure 2:**
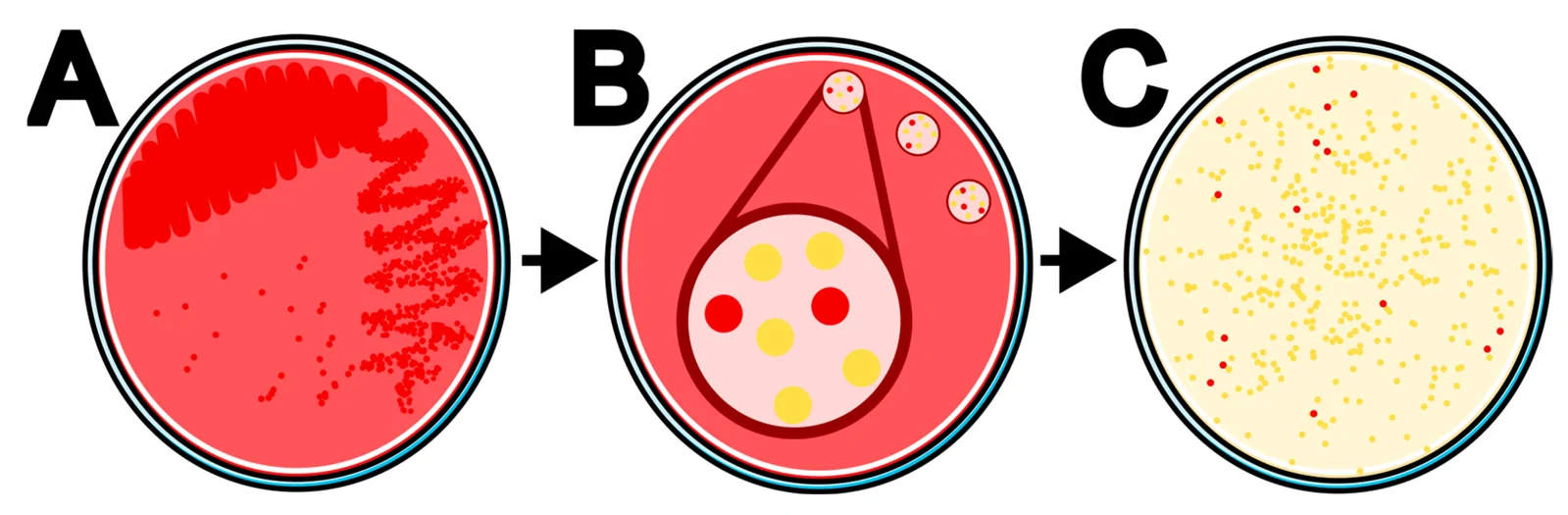
Schematic representation of the initial in vitro experiments. Colonies of *E. coli* J62 F*lac*::Tn3, with the plasmid-containing genes enabling lactose fermentation and ampicillin resistance (**A**), were mixed as a lawn with a high titer of a phage which specifically infects by attaching to plasmid-mediated sex pili. Following incubation (**B**), putative phage-resistant colonies growing within these plaques were streaked onto MacConkey agar (**C**). This resulted in a majority of yellow colonies which had lost the plasmid, and thus were phage-resistant, non-lactose fermenting and sensitive to ampicillin. A small number of lactose-fermenting (red) colonies retained the plasmid and were phage-resistant, but were no longer self-transmissible due to mutations in the *tra* operon which prevented pilus production.

Tectiviruses and Emesviruses have been used for plasmid-curing in vitro and in vivo. It seems likely that a small proportion of bacterial cells in any plasmid-containing culture lose their plasmid as a natural but infrequent random stochastic event regardless of the TA systems. In the presence of an antibiotic, these cells would normally die. However, in the absence of antibiotics and the presence of these phages, the plasmid-free cells show greater fitness and can outgrow the plasmid-containing parent cells (Figure 1). The presence of male-specific phages thus exerts a strong selection pressure against highly self-transmissible plasmids. The studies with MS2 and an *E. coli* F*lac* Amp^R^ indicated that MS2 was highly lytic for the bacterial strain, but that heavy secondary growth occurred and a high proportion of these phage-resistant cells have lost their ability to ferment lactose (Figure 2) and, when checked, they had lost the F*lac* Amp^R^ plasmid and had therefore become antibiotic-sensitive.

This has been shown to occur with the Tectivirus PRD1 and the RP4 plasmid, encoding resistance to kanamycin, tetracycline, and ampicillin, in *E. coli* and *Salmonella* Typhimurium [85], as well as with Emesvirus MS2, with an ampicillin resistance-encoding F*lac* plasmid in *E. coli* and *Salmonella* Enteritidis [86]. In both these studies the plasmids were highly self-transmissible, with >95% of bacterial cells producing the phage receptor. In both cases, relatively short periods of incubation of the AMR bacteria with the phage resulted in the plasmid-cured derivatives becoming dominant. A small proportion of phage-resistant mutants retained the plasmid but had ceased to be self-transmissible with mutations in genes in the transfer regions (*traJ* in the case of F [86] and *trbI*, *J* and *K* in the case of RP4 [87]. Some mutations were shown to be reversible as a result of insertion of tandem-repeats. For naturally highly transmissible plasmids this reversibility may have adaptive value and occur naturally to avoid phage susceptibility.

In vivo models included the larvae of the *Trichoplusia ni* moth [89] and young chickens [86]. In the former, the presence of the PRD1 phage had little effect on the absolute numbers of *Enterobacter cloacae* harboring RP4 or on the level of plasmid carriage in vivo compared with in vitro. In the chicken model, birds were infected with the AMR *S.* Enteritidis strain by contact, which was allowed to spread naturally through the population. Treatment with high-titer MS2 phage led to a reduction in bacterial numbers shed by the birds and, after a few days, complete replacement of the ampicillin-resistant, plasmid-carrying strain by an antibiotic-sensitive, plasmid-free derivative. In addition to plasmid-curing, both phages were shown to block or hinder conjugation [85,86].

The advantages of this approach are:

1. The phage may reduce or eliminate bacteria carrying highly transmissible plasmids and drive short-term evolution towards plasmid loss.
2. In contrast to standard phage therapy, phage-resistant mutants are sought and selected.
3. The phage can eliminate conjugation and therefore the spread of AMR between bacterial strains.
4. Phage specificity is related to the plasmid, not the bacterial species or strain which allows targeting of the same plasmid in different bacterial genera.

In addition, many male-specific phages:

5. Are obligately lytic, with the exception of the Inoviridae;
6. Have small genomes (e.g., Emesviruses, 3–4 kbp), limiting their capacity to acquire and carry virulence and other harmful genes;
7. Have demonstrated efficacy in the gastrointestinal tract.

In both the above sets of studies, the plasmids used were highly transmissible and therefore, with nearly every bacterial cell expressing sex pili, highly susceptible to phage- mediated selection of the plasmid-free bacterial cells. In reality, many wild-type plasmids show degrees of repression with transmission rates varying from 10^−2^ to <10^−8^ per donor cell. This mechanism allows bacteria harboring AMR and other plasmids to balance their capacity to transfer the AMR phenotype whilst ensuring that the majority of cells remain resistant to the phage. Colom et al. [86] showed that in vitro passage of a wild-type *E. coli* carrying a semi-repressed AMR plasmid in the presence of the MS2 phage displayed increases in plasmid loss from <0.1% to 4% of the bacterial population. Some clones, such as ESBL- and quinolone-resistant lineages of *E. coli* ST131, carry a highly repressed AMR plasmid and have become globally important [90,91]. AMR plasmids also integrate into the chromosome, which reduces their metabolic demand on the bacterial cell, sometimes with associated loss of transmissibility by truncation of the *tra* region [91]. This would also affect the susceptibility of these phages.

## 7. A Strategy to Resolve Plasmid Repression and Increase Phage Susceptibility

Plasmid repression is an important stumbling block/hurdle for this proposed technology to counter AMR, which will require further investigation and a new combined biotechnological approach.

**Figure 3:**
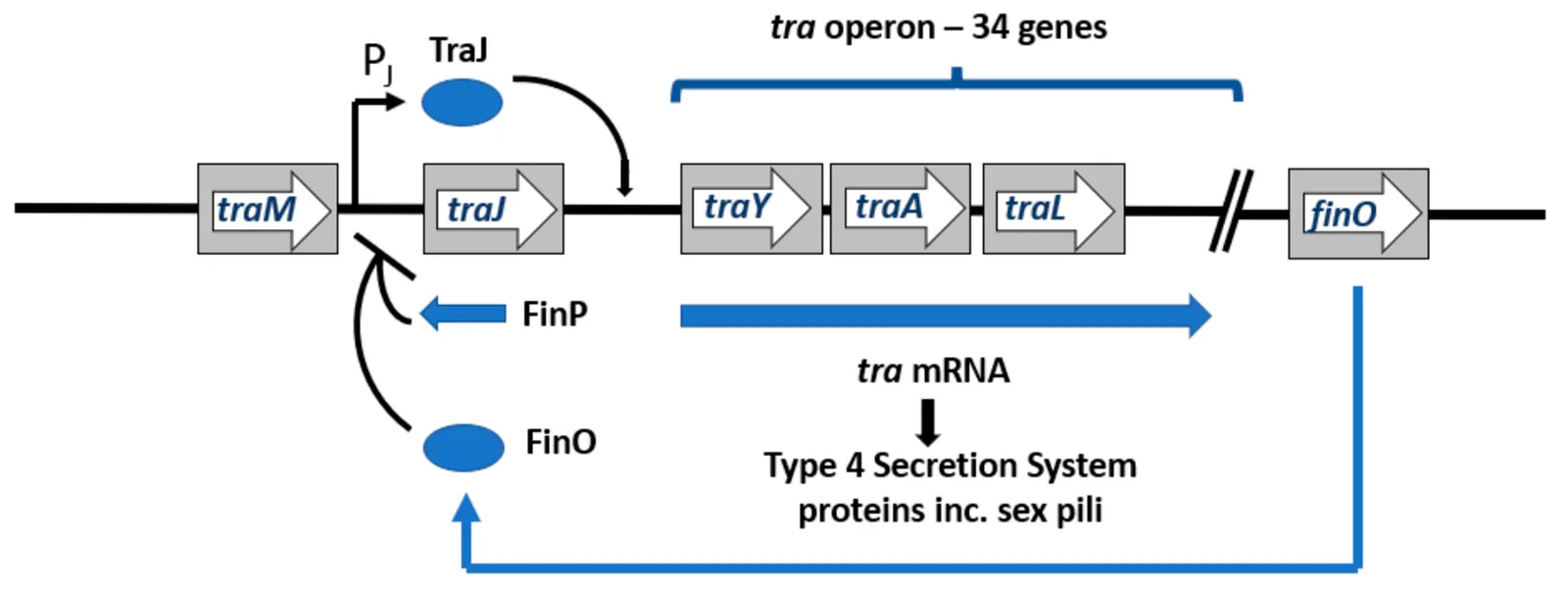
Mechanism of repression in Incompatibility group F plasmids. The FinP antisense RNA is transcribed in the reverse direction from the untranslated leader sequence of the *traJ* gene. This is able to block the production of the TraJ protein which normally activates the *tra* operon, resulting in the production of the sex pili and the whole plasmid transfer mechanism. The FinP RNA is susceptible to degradation by RNAseE but is stabilized by the FinO protein, and both block TraJ production, thereby repressing *tra* transcription. In the original F plasmid, transcription of *finO* is interrupted by an Tn*1000* insertion sequence and is completely de-repressed.

The method of repression of self-transmissibility is best understood in IncF plasmids [92] (Figure 3). A small antisense RNA FinP, transcribed from the complementary strand of the untranslated leader region of *traJ*, binds to the promoter region of *traJ* RNA. This prevents the translation of *traJ* which normally initiates the transcription of *traY,* the first gene in the *tra* operon. The FinO protein stabilizes FinP and its interaction with *traJ*, and prevents RNAseE degradation of FinP. The originally isolated F plasmid has an IS*3* insertion in *finO*, thereby naturally de-repressing *traJ* translation, conjugation and sex pilus production. Plasmid RP4 is also naturally de-repressed, which shows as high conjugation rates compared to many other clinically relevant plasmids [87].

The 3D crystalline structure of FinO has been determined, and active areas of the protein have been identified which might offer sites for small molecules to block the interaction with FinP [92,93]. Such a pharmaceutically active small molecule could be administered simultaneously with these phages to de-repress and increase phage susceptibility. Larger-scale searches for pharmaceutically active small molecules, perhaps identified by dynamic modeling of the potential interactions with the potential blocking sites in FinO and followed by large-scale screening, would likely be required.

The same investigations might be made with self-transmissible plasmids belonging to other Inc groups, although these and their modes of repression are much less well understood compared to IncF plasmids. In comparison with other approaches to reducing AMR and, specifically, plasmid-mediated AMR, the advantages of this approach are clear and further investigation is merited.

## 8. Bacteriophage and Virulence and Other Plasmids

Clearly, the interaction between sex pilus-specific bacteriophages and self-transmissible plasmids is a very interesting area of infection biology since, in addition to antibiotic resistance, transmissible plasmid-mediated characteristics include virulence determinants in *E. coli* such as colicin V, *E. coli* enterotoxin, the enteric adhesins K88 and K99, hemolysin, and the *Salmonella* virulence plasmid [94]. Interestingly, most of these plasmids belong to the IncF group and preliminary studies could easily be carried out using MS2.

More recently, the self-transmissible plasmid of emerging *Salmonella* Infantis (pESI) has caused extensive problems in the broiler chickens in Europe and elsewhere [95]. This megaplasmid frequently contains genes conferring resistance to multiple antimicrobials, along with metals and biocides, and appears to fall in Incompatibility group I [96].

This opens a potentially wide area of research on the relationship between phages, pathogens, AMR and virulence determinants. Intervention with sex pilus-specific bacteriophages may enable a closer study between AMR and virulence genes which can be found on the same plasmid [97] and may affect virulence stability and evolution.

## 9. Conclusions and Summary

Antimicrobial resistance is now one of the major existential threats to modern civilized life, and international institutions are exploring novel approaches to reducing resistance, including the use of phages. Although these bacterial viruses may be used to target bacterial cells, there are limitations to their use because of the high degree of specificity in terms of their interaction with the bacterial surface as the phage receptors. More exciting approaches include (i) the use of phagemids to disrupt bacterial or plasmid gene integrity and (ii) the use of phages that target the AMR mechanisms or the plasmids encoding resistance genes. The interaction between bacteriophages specific to the AMR phenotype in bacteria, especially the sex pili produced by self-transmissible plasmids, is a relatively novel area of research, but one which holds huge promise in modulating AMR. Research has shown that these can be highly effective in reducing levels of AMR plasmid carriage in vitro and in vivo and that there are several advantages to this approach. In silico analysis has suggested a way towards a pharmaceutical approach to increasing phage susceptibility for more repressed plasmids.

The interaction between these phages and other plasmid-borne attributes, including virulence and metabolic traits, in terms of evolution and epidemiology, could also be a new and exciting area of research.

## Data Availability

No new data were created or analyzed in this study. Data sharing is not applicable to this article.
